# The first investigation of Wilms' tumour atomic structure-nitrogen and carbon isotopic composition as a novel biomarker for the most individual approach in cancer disease

**DOI:** 10.18632/oncotarget.12521

**Published:** 2016-10-08

**Authors:** Katarzyna Taran, Tomasz Frączek, Anita Sikora-Szubert, Anna Sitkiewicz, Wojciech Młynarski, Józef Kobos, Piotr Paneth

**Affiliations:** ^1^ Department of Pathology, Medical University of Lodz, Poland; ^2^ Institute of Applied Radiation Chemistry, Lodz University of Technology, Poland; ^3^ Clinic of High Risk Pregnancy, Medical University of Lodz, Poland; ^4^ Department of Oncology and Paediatric Surgery, Konopnicka Memorial Hospital, Medical University of Lodz, Poland; ^5^ Department of Pediatrics, Oncology, Hematology and Diabetology, Konopnicka Memorial Hospital, Medical University of Lodz, Poland; ^6^ Department of Paediatric Pathology, Medical University of Lodz, Poland

**Keywords:** Wilms' tumour, stable isotopes, prognosis, anaplasia, personalized medicine

## Abstract

The paper describes a novel approach to investigating Wilms' tumour (nephroblastoma) biology at the atomic level. Isotope Ratio Mass Spectrometry (IRMS) was used to directly assess the isotope ratios of nitrogen and carbon in 84 Wilms' tumour tissue samples from 28 cases representing the histological spectrum of nephroblastoma. Marked differences in nitrogen and carbon isotope ratios were found between nephroblastoma histological types and along the course of cancer disease, with a breakout in isotope ratio of the examined elements in tumour tissue found between stages 2 and 3. Different isotopic compositions with regard to nitrogen and carbon content were observed in blastemal Wilms' tumour, with and without focal anaplasia, and in poorly- and well-differentiated epithelial nephroblastoma. This first assessment of nitrogen and carbon isotope ratio reveals the previously unknown part of Wilms' tumour biology and represents a potential novel biomarker, allowing for a highly individual approach to treating cancer. Furthermore, this method of estimating isotopic composition appears to be the most sensitive tool yet for cancer tissue evaluation, and a valuable complement to established cancer study methods with prospective clinical impact.

## INTRODUCTION

Wilms' tumour (nephroblastoma, WT) is one the best known cancers with well established prognostic parameters. Evaluation by light microscopy has proved to be the most useful method of WT examination in oncological practice. The histology of the lesion is currently a leading prognostic marker, which forms the basis of treatment selection in individual cases when considered together with disease stage. This current approach based on combined therapeutic regimens appropriate to the stage of disease advancement and risk, as estimated by microscopic examination, often give exceptionally good treatment results, with a current recovery rate of approximately 90% being noted. Although this figure is one of the best in Oncology, the results of WT treatment are still unsatisfactory, and unexpected problems still occur in the clinical course of cancer, including complete treatment failures [1]. The presence of these failures indicate that a certain level of tumour biology defies standard ways of evaluation.

Isotope Ratio Mass Spectrometry (IRMS) is a highly-precise method of distinguishing biological material via assessment of isotope composition. Although this method originally found use in Archaeology and the Earth sciences, the measurement of isotope ratio is frequently harnessed in numerous divergent scientific disciplines with its scope of use constantly extending to new areas. It is commonly exploited to study the mechanism of biochemical reactions [2]. The molecules involved in biochemical processes are subject to changes in isotopic composition due to the presence of varying numbers of isotopic species (isotopologues). This is referred to as the isotope effect, and the resulting change of isotopic composition is recognized as isotopic fractionation.

Changes of isotopic ratio have been already confirmed in non-cancerous pathological conditions in humans [3, 4], and in the course of cancer [5, 6, 7]; however, these studies were based on samples of patient serum, hair or breath, rather than the assessment of the abnormal tissues themselves. An investigation of cancer cell lines using an IRMS and an Elemental Analyzer coupled to a spectrometer was found to be able to effectively characterise the origin of diseased or healthy cells, as well as their condition [8].

The isotopic composition of cancer tissue growing *in vivo* seems to be the last unknown area of its biology. It may also represent a potential source of data needed for the assessment of the most individual characteristics of cancer and complements those at the microscopic or molecular levels. Recently, a few preliminary studies have examined isotope ratios directly in tumour tissues [9, 10, 11, 12] with promising indications that isotopic composition may facilitate the classification of patients into risk groups [11, 12]. However, these previous studies have not covered all the prognostic parameters appropriate to the cancer type, which constitute the existing base for the choice of treatment protocol and prognosis in individual cases. Although stable isotope ratio measurement is not an established procedure in Oncology, its proven quality and credibility suggests that this method could be especially valuable in studies with prospective clinical impact. Comparison of results of isotopic analysis and well established prognostic parameters seems to be the simplest way to preliminary evaluate the potential value of isotope ratio assessment for personalized medicine and clinical practice.

The present study represents the first direct assessment of the isotopic composition of tumour tissues taken from a spectrum of histological types of one tumour. The isotopic composition of the cancer tissue is compared with well-established prognostic parameters to reveal a novel biomarker for the most individual approach in cancer disease, intended to avoid treatment problems and failures.

The choice of nitrogen and carbon as the elements to be examined in tumour tissue was guided by the fact that they play a key role in the emergence and sustenance of cell metabolism and life, during such processes as cell growth and division, which despite being important for cancer biology, have yet to be subjected to any such cancer tissue research. In addition, the isotopic composition of these two elements can be now examined on a routine basis at a level of precision sufficient for diagnostic applications.

## RESULTS

### Natural abundance of ^15^N and ^13^C in Wilms' tumour tissues and in a normal kidney cortex

In 84 performed measurements in WT tissues (i.e. three separate estimations of nitrogen and carbon isotope composition for each tumour), the values of δ^15^N (vs. air N_2_) ranged from 2.45% (minimum) to 12.21% (maximum) and the values of δ^13^C (vs. V-PDB) from −23.59% (minimum) to −17.77% (maximum). The normal kidney cortex tended to be ^15^N-depleted and ^13^C-enriched in comparison with cancer tissues (see Table 1).

**Table 1:** The summary of isotopic composition of the examined cancer (WT group) and healthy tissues (normal kidney cortex, n = 2)

Tissue type	δ^15^N Mean (SD) (%)	δ^13^C Mean (SD) (%)
Wilms' tumour group	8.23 (± 1.51)	−22.24 (± 1.27)
Normal kidney cortex	7.61 (± 0.18)	−21.97 (± 1.17)

### Isotopic picture of WT histological types

The division of WT group into histological types according to the International Society of Paediatric Oncology (Société Internationale d'Oncologie Pédiatrique, SIOP) revealed differences in isotope ratio for the two elements. The mean values of δ^15^N and δ^13^C in the examined WT histological types were highly variable, observed differences ranging from 1.21% (minimum) to 9.11% (maximum).

The low and high risk groups were represented by one histological type in our studies, with the mean value of δ^15^N being 6.79% (± 1.52) and δ^13^C being 20.98‰ (± 1.27) in intermediate-risk tumours. No statistically significant differences in nitrogen and carbon delta values were observed between histological types or risk groups.

**Photo 1:**
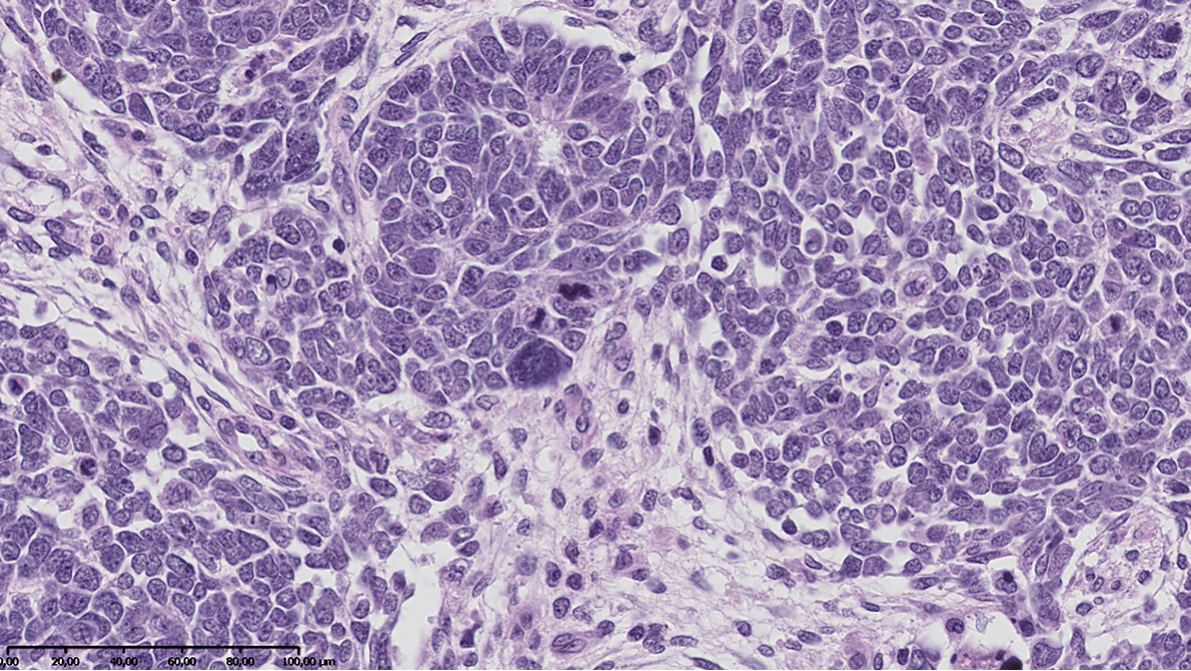
Anaplasia in blastemal type of nephroblastoma – abnormalities of cell nuclei observed in microscopic examination were accompanied by lower values of δ^15^N and δ^13^C in comparison with other blastemal WTs (H&E, oryg. magn. 200×)

**Photo 2:**
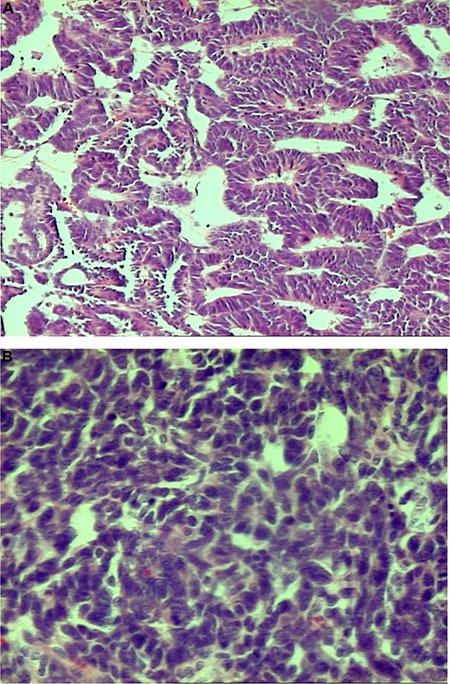
Nephroblastoma – well-differentiated epithelial type, H&E, oryg. magn. 100× (2A-top) presented higher values of δ^15^N and δ13C than the poorly-differentiated type, H&E, oryg. magn. 200× (2B-bottom)

**Figure 1:**
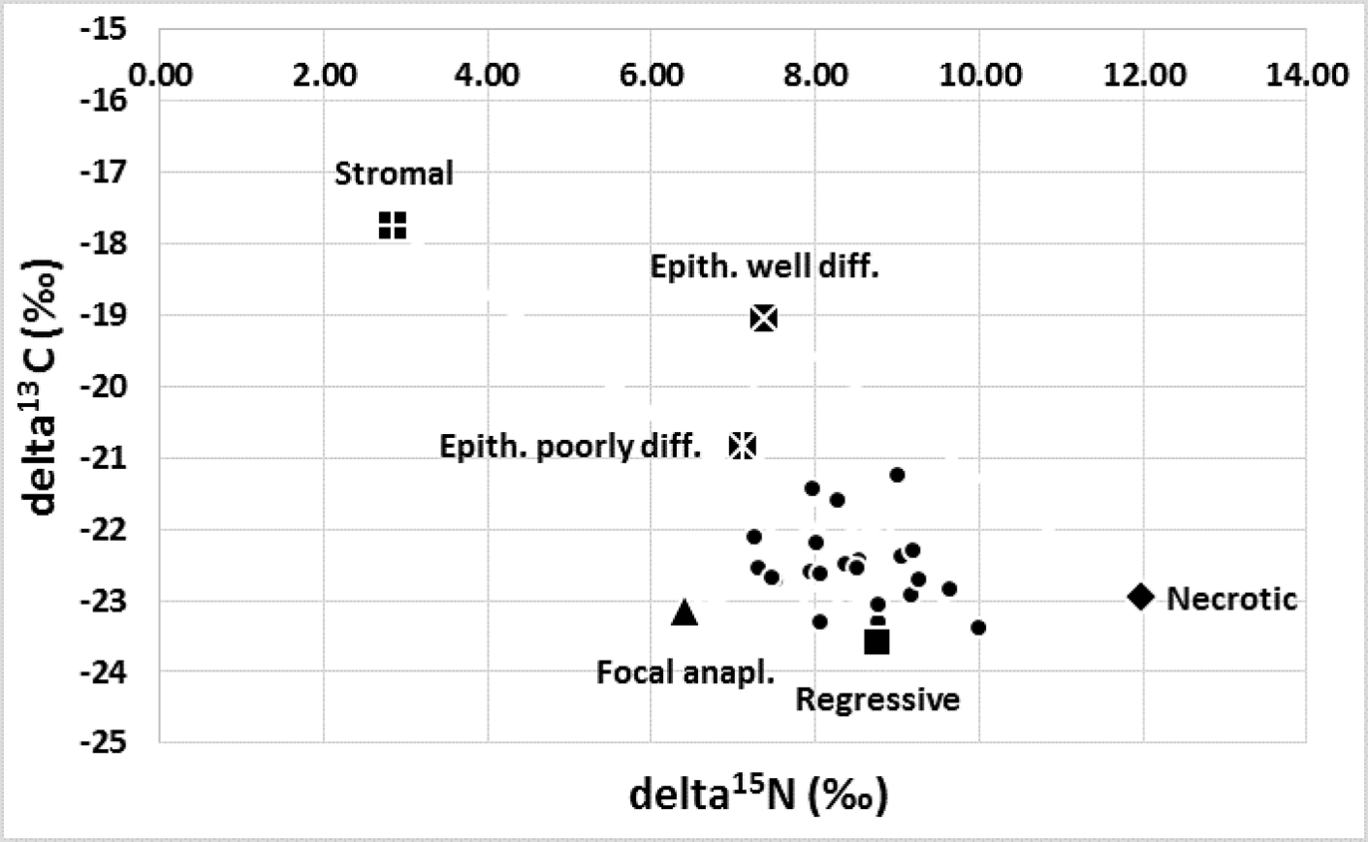
Variation in the isotopic composition of tumours in the WT group (*n* = 28, δ^15^N ±1.51, δ^13^C ± 1.27) The maximal abundance of ^15^N was observed in a completely necrotic WT case and a minimal abundance in the stromal type. Maximal abundance of ^15^C was found in stromal WT and minimal abundance in one of the regressive cases. The composition of the C and N isotopes varied between epithelial tumours with different levels of differentiation. The most intriguing results were related to the presence of focal anaplasia (see detailed description in the text).

A 1.81% difference was observed in the mean value of delta ^15^N between a case of Blastemal WT with the features of focal anaplasia (Photo 1) and those without evidence of anaplasia. Similarly, a difference of 3.01% was found for delta ^13^C between poorly-differentiated epithelial WT and well-differentiated epithelial WT (Photo 2A and 2B). The isotopic studies in the WT group are summarised in Table 2 and Figure 1.

**Table 2:** Nitrogen and carbon isotopic composition in Wilms' tumour histological types

Nephroblastoma type	Risk according to SIOP	δ^15^N Mean (SD)(%)	δ^13^C Mean (SD)(%)
Completely necrotic nephroblastoma	Low	11.97 (± 0.21)	−22.96 (± 0.32)
Nephroblastoma epithelial type	Intermediate	7.52 (± 0.49)	−21.06 (± 2.13)
Nephroblastoma well-differentiated epithelial type		7.59 (± 0.67)	−22.07 (± 1.74)
Nephroblastoma poorly-differentiated epithelial type		7.39 (± 0.12)	−19.06 (± 0.15)
Nephroblastoma stromal type	Intermediate	2.86 (± 0.35)	−17.77 (± 0.30)
Nephroblastoma mixed type	Intermediate	8.51 (± 1.04)	−22.44 (± 0.78)
Nephroblastoma regressive type	Intermediate	8.26 (± 0.59)	−22.64 (± 0.53)
Nephroblastoma blastemal type	High	7.63 (± 1.40)	−22.87 (± 0.31)
Nephroblastoma blastemal type without features of anaplasia		8.24 (± 1.32)	−22.72 (± 0.26)
Nephroblastoma blastemal type with focal anaplasia		6.42 (± 0.32)	−23.16 (± 0.21)

### Isotopic picture of Wilms' tumours in subsequent stages of cancer disease

**Figure 2:**
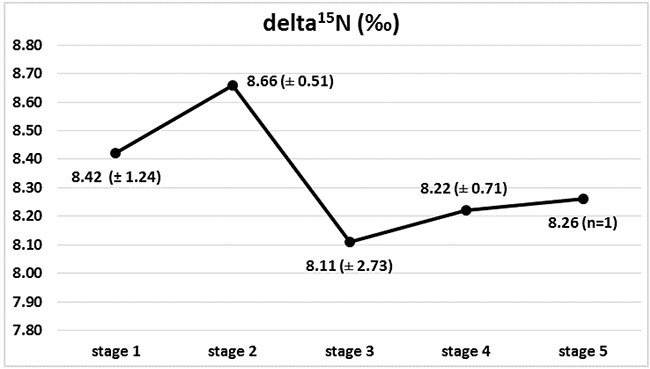
Isotopic composition of nitrogen (delta ^15^N) in Wilms' tumour group in subsequent stages of disease, with marked ^15^N depletion between stages 2 and 3

**Figure 3:**
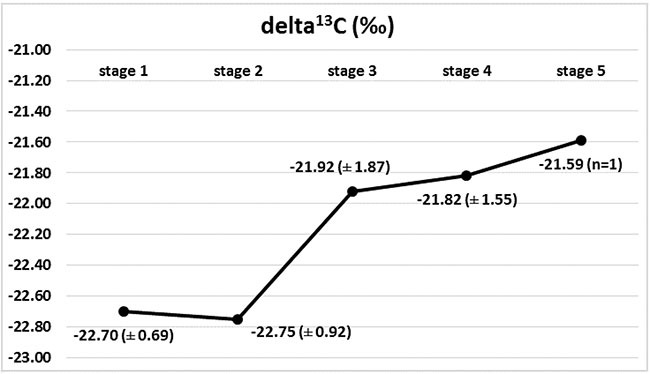
Isotopic composition of carbon (delta ^13^C) in Wilms' tumour group in subsequent stages of disease, with marked ^13^C enrichment between stages 2 and 3

**Figure 4:**
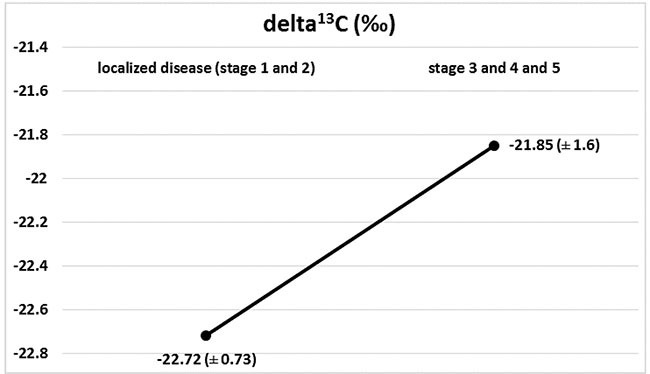
Isotopic composition of carbon (delta ^13^C) in localized disease (stages 1+2) and in progression (stages 3 + 4 + 5), (*p* = 0.052)

Marked changes in the ratios of the C and N isotopes were observed in tumour tissues at the course of cancer disease. The greatest change in nitrogen isotope composition (depletion) was observed between stages 2 and 3, and it was accompanied by the most distinct carbon enrichment, Figures 2 and 3. The difference in carbon isotope ratio between localized tumours (stages 1 and 2) and more advanced cancer (stages 3, 4 and 5) approached statistical significance (*p* = 0.052), Figure 4.

## DISCUSSION

An important novel aspect of our study is that it describes the first evaluation of the isotopic composition of WT at the atomic level in relation to well-established prognostic parameters. In addition, different ratios of nitrogen and carbon isotopes between normal and cancer tissues were identified. We have observed differences of isotope ratio in the WT group and that they reflected both the widely-accepted prognostic markers, histological type and stage of the disease. It suggests that the modification of the metabolism specific to each histological WT type gives rise to a modified isotope fractionation pattern. While it was reported that variations in the isotopic composition were associated with biochemical processes in cancer, this phenomenon has previously only been observed *in vitro* in cancer cell lines [8]. Our present study demonstrates its presence in tumour tissues growing *in situ*, and indicates that in living organisms, variations in the composition of nitrogen and carbon isotopes reflect cancer cell metabolisms and are characteristic of a tumour, as well as its histological type. More, they characterize the degree of disease progression with potential prognostic impact.

The first part of our findings, the great variation in the isotopic ratios of nitrogen and carbon, confirmed the complexity of WT on the atomic level and corresponded to the standard methods of profiling its histogenesis. The fact that the delta values of both elements appeared to differ according to the histological components of WT, corresponds to the distinct biological differences of WT types documented in the literature. It was previously reported that protein expression in WT components varies to a great degree [13, 14, 15] and shows various prognostic values [16]. This may indicate that the differences in isotopic ratios observed in WT cells can be attributed to amino acid metabolism and variations in the metabolic pathways used in their synthesis, as has been observed in other types of cells [17, 18].

The differences may reflect the complex chemical reactions occurring during WT cell growth and differentiation. Previous studies have noted differences in isotopic ratio present between different tissues [19] and that these tissues have unique amino acid compositions with amino acids that vary in their isotopic ratios [20, 21]. In our study, the WT tumours with significant differentiation, for example stromal WT, presented extreme isotope ratios and this may be related to the changes in its amino acid composition occurring during the processes of tumour cell differentiation. It is also known that some types of WT develop from pre-existing lesions. The fate and metabolic pathways of nephrogenic rests are very complex, as well as biology of tumours that develop from them [22]. Changes in amino acid metabolism are known to occur in relation to metabolic rate. However, it could also reflect a change in the proteome.

It is also possible that the existence of various directions of differentiation, the presence of up to three possible components (blastemal, epithelial and stromal), and complicated nature of WT pathogenesis may be responsible for the apparent incompatibility of isotopic studies results. They may also be responsible for the lack of any statistical significance when assessed as a group.

It is worth stressing that our findings are the first to confirm the exceptional value of currently used WT histological classifications at the atomic level. The distinct differences observed in isotopic ratios among WT histological types clearly demonstrate the complexity of WT biology. In addition, the small differences visible among less differentiated tumours highlight the common primary origin of all WT cells and their similar metabolic pathways.

The most surprising discovery in our studies was the relationship between nitrogen and carbon isotope composition and the course of cancer progression. It was found that the isotope ratio of elements reflecting cancer cell metabolism does not remain stable throughout the course of the disease.

It is intriguing that the most marked changes of isotope ratio of both elements (nitrogen depletion and carbon enrichment) appeared together and at the key moment of the development of cancer disease regarding its dissemination and breakout in prognosis. It is difficult to interpret these findings as the isotopic profile of C and N during the cancer progression has not been previously investigated, and the knowledge of normal cells is related to many very special circumstances, such as the availability of elements in the natural setting and their assimilation, as well as the presence of internal and external factors including temperature, age, tissue type and species, the presence of hormones and the rate of metabolism [3, 23–26]. However, studies on other living tissues indicate that nitrogen depletion corresponds to an increase in tumour biomass. This has been observed in other anabolic processes which are characterised by a decline in the value of δ^15^N, in contrast to cell catabolism, which is referred to nitrogen enrichment [27–33].

The second part of obtained results far exceeded our expectations. It was most probably achieved due to the very high precision allowed by the apparatus, which is not available in routine methods of tumour tissue evaluation. Differences were observed in the isotopic composition of all of the most intensively investigated and/or the most enigmatic parts of WT: the biology of blastema, the features of anaplasia and quite surprisingly, the nature of epithelial component. The differences were found in very narrow groups of WT tumours, free of limitations from histogenesis, resulting in high reliability of the obtained isotope ratios.

The typical blastemal component presented the highest values of δ^15^N among all the examined WT cells. In contrast, blastemal WT with anaplasia (focal type) was associated with an extreme isotopic ratio and much lower values of δ^15^N. Anaplasia is an independent prognostic factor in WT, according to the definition introduced by the National Wilms' Tumour Study and subsequent modifications [34–36]. It is considered to be a negative prognostic marker when it appears as a diffuse feature or when it is recognized in an advanced stage of the disease; in such situations, treatment intensification is necessary [37, 38], due to the greater resistance of the tumour to chemotherapy [39, 40].

In our study, WT with focal anaplasia demonstrated the lowest nitrogen isotope ratio. That finding may indicate that a high proliferation rate, which characterizes tumours with features of anaplasia and results in an aggressive and commonly fatal course of the disease, may be related to and identified by a low nitrogen isotopic ratio. This is probably due to the fact that this element is required for proliferation by proteins, nucleoproteins and nucleic acids. Our findings may also indicate that isotope ratio measurements can reveal the most subtle, even only focal, signs of abnormalities in cell biology, a potential source of treatment problems or failures. It cannot be excluded also, that these extremely low nitrogen isotope ratios may be novel biomarkers of the worst prognosis.

An unexpected finding was the difference in carbon isotopic composition of well- and poorly-differentiated epithelial WT. Currently the level of differentiation of epithelial tumours does not influence the prognosis, and all are classified in the intermediate risk group. However, in the prior histological division, less differentiated epithelial WT was related to a worse prognosis. The observed differences in the isotope composition of epithelial WT may be a potential explanation of discrepancies and conflicting results in studies related to the surprising biology of this unusual WT type [41, 42] and may indicate the presence of different metabolic pathways between well- and poorly-differentiated epithelial WT.

### Future perspectives

This analysis of isotopic composition of cancer tissues represents a novel area of research on biomarkers in cancer; it is also a very sensitive method with potential value for therapeutic strategies as a part of personalized medicine, which may help minimize the implementation of sub-optimal regimes, treatment failures and unjustified increases in therapeutic expenses.

Finally, being the first evaluation of isotopic composition of tumour tissue in relation to well-established prognostic parameters, our studies also call for research in this newly-discovered area of cancer biology with potential clinical impact.

## MATERIALS AND METHODS

Permission was given for the study by the Bioethics Committee of the Medical University of Lodz (RNN/99/13/KE). A total of 84 Wilms' tumour tissue samples from 28 cases were selected for IRMS studies. They were obtained from 14 males and 14 females from Lodz region, Poland, aged from 0.1 months to 132 months (mean 45.0, SD ± 38.18). Metastases were observed in four cases and recurrences in three. No children died due to progression of the disease during the study. Due to the ethical reasons, the samples of normal kidney (cortex) were obtained in post mortem examination of two child victims of car accidents from the same region as the cancer patients: both were males, aged 84 and 96 months.

### Histological procedures

In standard histological procedures, all the abnormal tissues were reviewed routinely by two pathologists and assessed according to the current ‘Revised International Society of Paediatric Oncology (SIOP) working classification of renal tumours of childhood’. The diagnosis was confirmed with the use of a panel of immunohistochemical stains typical for small round cell tumours including WT-1(+) antibodies. Microscopic evaluation of the kidney cortex did not reveal any abnormalities.

### ^15^N and ^13^C abundance by continuous flow isotope ratio mass spectrometer (CF-IRMS) coupled with elemental analyzer for simultaneous carbon-nitrogen-sulphur (NCS) analysis

A part of the materials intended for CF-IRMS studies was taken after surgery or autopsy and was not routinely fixed but frozen and kept at −80°C until assessment in order to preserve their eligibility for isotope ratio evaluation.

For the purposes of CF-IRMS measurements, samples of 5 ± 1mg were prepared from frozen tissues. They were placed in 12.5 × 5 mm tin capsules, and dried in a vacuum for five hours at room temperature. Around 1 mg of vanadium pentoxide was added to each sample as a sulphur oxidation catalyst, and capsules were folded carefully the same way as described by Taran [9]. Next, the isotope ratios of nitrogen ^15^N/^14^N and carbon ^13^C/^12^C were measured in triplicate for each tumour case with the use of a Sercon 20–22 Continuous Flow Isotope Ratio Mass Spectrometer (CF-IRMS) coupled with a Sercon SL Elemental Analyzer for simultaneous carbon-nitrogen-sulphur (NCS) analysis as described by Fry [43]. The ^15^N and ^13^C abundance was showed as delta (δ) values (in parts per mil, ‰), relative to international standards for nitrogen (atmospheric, air N_2_) and carbon (Pee Dee belemnite, V-PDB) according to the formula: δ (‰) = (R_sample_/R_standard_−1) * 1000, where R_sample_ and R_standard_ are heavy/light isotopic ratios for the sample and international standard, respectively.

A sample of in-house standard thiobarbituric acid calibrated against international standards (δ^15^N = −0.23 (air N_2_), δ^13^C = −28.35 (V-PDB)) was used as the primary reference. The secondary standard material was glutamic acid (δ^15^N = 4.8 (air N_2_), δ^13^C = −27.3 (V-PDB)) received from the CEISAM laboratory, University of Nantes. For both standard materials; thiobarbituric acid and glutamic acid, 3 ± 0.5 mg were placed in 12.5 × 5 mm tin capsules (with 1 mg of vanadium pentoxide in the case of thiobarbituric acid).

### Statistics

Statistica 12.5 software (Statsoft, Tulsa, OK, USA) was used for statistical analysis. Descriptive statistics of the measurable parameters were calculated and presented as mean with standard deviation (SD). The obtained values of isotope ratio of nitrogen and carbon were analyzed in relation to widely-accepted prognostic markers, WT histological type and stage of the disease. The normality of the data was determined by the Shapiro-Wilk W-test, and nonparametric methods of analysis were chosen: the Mann-Whitney *U-test* and the Kruskal-Wallis test. The results were considered statistically significant at *p* ≤ 0.05.

## CONCLUSIONS

Our findings confirm for the first time that the evaluation of the isotopic composition of cancer tissue reflects biochemical processes and its abnormal metabolisms, which influence the course of the disease. Furthermore, our findings suggest that the measurements of nitrogen and carbon isotope ratio in WT tissue can reveal the most critical moment of cancer dissemination and, together with the established prognostic parameters, may be used to separate the cases with the most doubtful clinical outcome, which need a more individual approach.
